# Vδ2^+^ T Cells—Two Subsets for the Price of One

**DOI:** 10.3389/fimmu.2018.02106

**Published:** 2018-09-25

**Authors:** Martin S. Davey, Carrie R. Willcox, Stuart Hunter, Ye Htun Oo, Benjamin E. Willcox

**Affiliations:** ^1^Cancer Immunology and Immunotherapy Centre, Institute of Immunology and Immunotherapy, University of Birmingham, Birmingham, United Kingdom; ^2^Centre for Liver Research and National Institute for Health Research (NIHR), Birmingham Biomedical Research Centre, Institute of Immunology and Immunotherapy, University of Birmingham, Birmingham, United Kingdom; ^3^University Hospital of Birmingham NHS Foundation Trust, Birmingham, United Kingdom

**Keywords:** T-cells, innate, adaptive, phosphoantigens, cytomegalovirus

Vδ2^+^ T cells are a relatively well characterized lymphocyte subset, and are predominant among the γδ T cell compartment in human peripheral blood. Known to increase post-natally as a proportion of peripheral blood γδ T cells in early life (1, 2), they feature a dominant but not universal Vγ9^+^ Vδ2^+^ usage (3), and mostly recognize low-molecular-weight pyrophosphate antigens (pAg), which can be either derived from the host mevalonate pathway (isopentenyl pyrophosphate, IPP) or microbially generated via the non-mevalonate pathway ((E)-4-Hydroxy-3-methyl-but-2-enyl pyrophosphate, HMB-PP) (4). In addition, although recent work from our group provides some evidence for selective maturation during life (5), it also clear that the Vδ2^+^ repertoire is dominated both in neonates and adults by a semi-invariant Vγ9^+^ Vδ2^+^ TCR repertoire, including public Vγ9 clonotypes, consistent with an innate-like paradigm involving pre-programmed recognition of pAgs from birth (5, 6).

However, our recent data (5) suggest that in addition to pAg-reactive Vγ9^+^ Vδ2^+^ T cells, the Vδ2^+^ T cell compartment harbors a distinct subset of Vγ9^neg^ T cells, which adopt a highly divergent immunobiology from their semi-invariant Vγ9^+^ colleagues. The existence of Vγ9^neg^ Vδ2^+^ T cells has been recognized for a number of years. Data from cord blood indicated that the Vδ2 chain can associate with a variety of different chains other than Vγ9 (7). However, whether these cells generally persist into adulthood was unclear, as was their role and significance. Some observations have hinted at both persistence and significance. In a rare form of autoimmune polymyositis, highly clonal Vγ9^neg^ Vδ2^+^ T cells were observed to infiltrate into muscle and peri-muscular zones and destroy muscle fibers (8, 9). Furthermore, a patient with Felty's syndrome, which is characterized by leukopenia and splenomegaly in the context of seropositive rheumatoid arthritis, was found to have an expanded population of Vγ9^neg^ Vδ2^+^ T cells in peripheral blood that was capable of producing TNFα in response to *in vitro* CD3 stimulation (10). In addition, expanded Vγ9^neg^ Vδ2^+^ T cell clonotypes were observed in a single individual following stem cell transplantation (SCT), and a single healthy control (11). Furthermore, a recent study noted the presence of Vδ2^+^ TCR clonotypes within TCR repertoire analyses of the Vγ9^neg^ compartment, and detected variable numbers of Vγ9^neg^ Vδ2^+^ T cells by flow cytometry in healthy adults and chronic HCV patients (12). However, despite these efforts, it was unclear if such Vγ9^neg^ Vδ2+ T cells represented isolated clones in certain individuals, or reflected a common and immunophenotypically and/or functionally distinct T cell subset.

Now a recent study from our own laboratory establishes that Vγ9^neg^ Vδ2^+^ T cells commonly persist into adulthood, typically at low levels, and that they represent a previously unrecognized adaptive T cell subset (Figure 1). The study provides both immunophenotypic and TCR repertoire-based evidence strongly supporting an adaptive biology, identifies a microbial stimulus the subset can respond to, establishes they can access solid tissues (specifically the liver), and also describes flow cytometry-based methods for their routine detection (5). Vγ9^neg^ Vδ2^+^ T cells typically adopted a naïve phenotype in peripheral blood, accompanied by a diverse and highly private TCR repertoire (including Vγ2-8 chains), but occasionally displayed a differentiated, clonally expanded T_effector_ phenotype. In addition, Vγ9^neg^ Vδ2^+^ TCRγ and TCRδ CDR3 regions lacked motifs associated with pAg recognition, and unlike Vγ9^+^ Vδ2^+^ T cells did not utilize JγP, instead preferentially utilizing Jγ1/2 or JγP1. Consistent with these observations and with reports that Vγ9^+^ Vδ2^+^ reactivity to pAg is dependent upon both Vδ2 and Vγ9 chains (15), the Vγ9^neg^ Vδ2^+^ subset was not pAg reactive. These new findings suggested a close parallel between the immunophenotypic features of the Vγ9^neg^ Vδ2^+^ subset and those of Vδ1^+^ T cells (16, 17), and highlighted key differences with the Vγ9^+^ Vδ2^+^ T cell subset.

**Figure 1:**
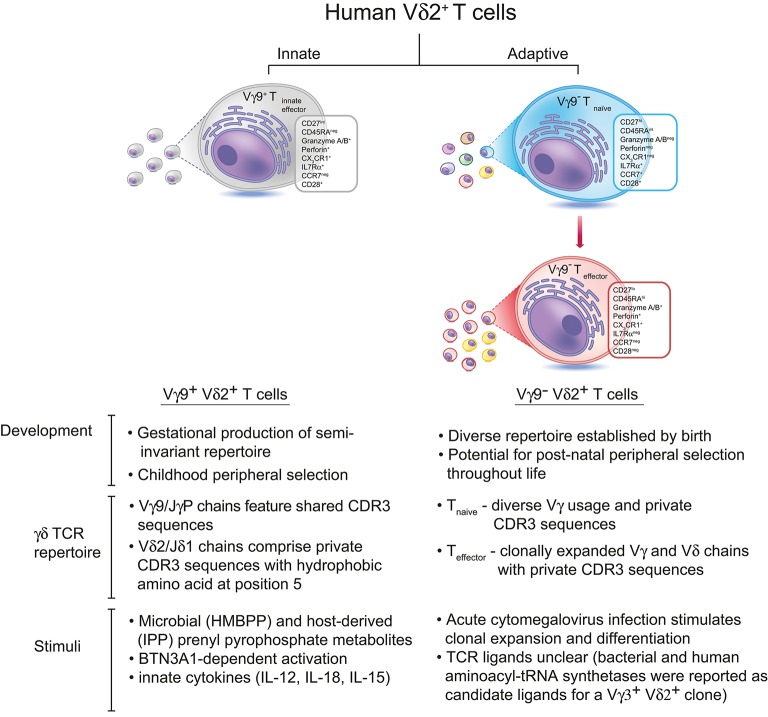
There are two subsets of Vδ2^+^ T cells in human peripheral blood. The predominant Vγ9^+^Vδ2^+^ subset is generated during gestation, expresses Vγ9 chains with public CDR3 sequences, and undergoes peripheral selection and polyclonal expansion during childhood to become pAg-reactive “innate-like” effector cells (13). In contrast, Vγ9^neg^Vδ2^+^ T cells express Vδ2 paired with various Vγ chains bearing private CDR3 sequences, and these cells circulate in the peripheral blood as naïve T cells until they encounter a specific antigenic challenge (which can include, but is likely not limited to, CMV infection). Antigen-specific Vγ9^neg^Vδ2^+^ T cells undergo clonal expansion and differentiate into effector T cells, similar to Vδ1^+^ T cells. The antigens recognized by Vγ9^neg^Vδ2^+^ T cells remain unknown, although bacterial and human aminoacyl-tRNA synthetases have been identified as candidate antigens for a single Vγ3^+^Vδ2^+^ T cell clone (14).

What immune challenges might the Vγ9^neg^ Vδ2^+^ subset respond to? The apparently similar immunobiology of Vγ9^neg^ Vδ2^+^ and Vδ1^+^ T cells suggested viral infection as a likely candidate, given that in response to CMV Vδ1^+^ T cells can increase in number (18, 19), and undergo clonotypic expansion (11), and that they may also respond to other viruses (20, 21). This was confirmed by the observation that after acute CMV, Vγ9^neg^ Vδ2^+^ T cells can transition from from a CD27^hi^ naïve-like phenotype to CD27^lo/neg^ effector-like populations, alongside clonal expansion (5). This finding extends previous studies of Ravens (11) and Davey (16) by providing clear confirmation that an individual microbial stimulus can not only induce TCR clonotypic expansion but also concomitant adaptive differentiation from T_naive_ to T_effector_ status. Of strong significance is the observation that, for both Vγ9^neg^ Vδ2^+^ T cells and Vδ1^+^ T cells, this transition is marked by upregulation effector/cytotoxic markers including Granzyme A, CD16, as well as downregulation of lymphoid homing markers strongly expressed on naïve populations such as CCR7, and upregulation of the peripheral homing marker and chemokine receptor CX3CR1 (5, 16). Also, the CDR3 sequences of clonotypically expanded TCRs observed in different individuals were diverse, as for Vδ1^+^ expansions, contrasting with the high level of Vγ9 TCRγ publicity observed within the Vγ9^+^ Vδ2^+^ T cell repertoire. Another key feature of Vγ9^+^ Vδ2^+^ T cells, namely their predominant peripheral blood localisation, was also compared for Vγ9^neg^ Vδ2^+^ T cells, in the context of human blood and liver samples. Tellingly, whereas the Vδ2^+^ T cell compartment as a whole was preferentially enriched in peripheral blood, the Vγ9^neg^ Vδ2^+^ subset was preferentially enriched in human liver relative to peripheral blood. This result indicates that the Vγ9^+^ Vδ2^+^ and Vγ9^neg^ Vδ2^+^ subsets are not only distinguished by their TCR repertoire, immunophenotype, and responsiveness to distinct microbial challenges, but also by their homing properties, and further strengthens parallels with Vδ1^+^ T cells, which share the ability to respond to CMV (11), and are also preferentially enriched in solid tissues such as the liver (22).

The Vδ2^+^ T cell compartment therefore includes both innate-like and adaptive subsets, which have a very different immunobiology to each other. In addition, the fact that Vγ9^neg^ Vδ2^+^ T cells appear to adopt a very similar overall biology to Vδ1^+^ T cells (16, 17) is intriguing, and suggests the existence of an adaptive γδ paradigm in humans that at least these two distinct subsets (Vδ1^+^ and Vγ9^neg^ Vδ2^+^ T cells) appear to exhibit. Furthermore, the fact that both Vγ9^neg^ Vδ2^+^ and Vδ1^+^ T cell expansions display an effector phenotype, are relatively long-lived, and in the case of Vδ1^+^ T cells display a far quicker response to TCR stimulation than their naïve counterparts, suggests their potential to contribute to immunoprotective effector memory responses following initial pathogen exposure (5, 16, 17). Clearly many questions regarding this paradigm are still unresolved—such as how clonal expansion is initiated, about the underpinning transcriptional control mechanisms, and also crucially regarding the nature of TCR ligands that trigger such clonal expansions, both in blood and solid tissues. However these recent studies establish clonotypes and highlight clinical scenarios with which to answer these questions.

Importantly, these recent findings establish the antibodies required for reliable flow cytometry-based identification of this new subset, thereby paving the way for investigation of Vγ9^neg^ Vδ2^+^ T cells in a wider range of peripheral tissues. Conceivably, due to the inability of some Vδ2-specific mAbs to detect Vγ9^neg^ Vδ2^+^ T cells (5), the presence of this subset could have been overlooked in some previous studies. Additionally, while Vγ9^neg^ Vδ2^+^ T cells clearly clonally expand in response to CMV, as do Vδ1^+^ T cells, the full range of pathogens they respond to is unclear, and the suspicion is that, as for Vδ1^+^ T cells, a wider range of pathogens is likely to be relevant. Similarly, while current studies have been restricted to studying the subset in blood and the liver, there are likely to be additional tissues in which Vγ9^neg^Vδ2 T cells are present and can mount such responses.

These recent findings raise many questions regarding Vγ9^neg^ Vδ2^+^ T cells, such as why, given the evidence that the subset responds to acute CMV within peripheral blood, do most individuals chronically infected with CMV harbor peripheral blood Vγ9^neg^ Vδ2^+^ T cell populations that are naïve. Despite the observation that Vγ9^neg^ Vδ2^+^ T cell clonotypes can persist for 5 years (at least in immunosuppressed individuals), one possibility is that the kinetics of their differentiation favor a limited duration for Vγ9^neg^ Vδ2^+^ T cell responses. Notably, the Vγ9^neg^ Vδ2^+^ T_effector_ response can become increasingly focused on fewer TCR clonotypes over that time period (5). It is therefore possible that terminal differentiation and subsequent apoptosis focuses and ultimately eliminates such T_effector_ responses, depending on their duration since initial CMV infection. Another, not mutually exclusive possibility, is that only a limited proportion of individuals are able to respond to primary infection in the first instance. This is consistent with our recent observations in acute CMV infection, where one of three patients who developed acute CMV did not appear to mount a Vγ9^neg^ Vδ2^+^ T cell response (5), at least in peripheral blood. One caveat is that recent work on hepatic Vδ1^+^ T cells has shown that while some expanded clonotypes are present in both blood and the liver, others are restricted to the liver and display a distinct phenotype suggestive of hepatic residency (22). If this same principle applies to the Vγ9^neg^ Vδ2^+^ T cell subset, it is conceivable that some individuals mount a response restricted to peripheral tissue compartments but undetectable in blood. A third, non-mutually exclusive possibility is that the emergence of a Vγ9^neg^ Vδ2^+^ T cell response, or lack thereof, is dependent on contributions of other arms of the immune system, in keeping with the idea that γδ T cell responses may be exacerbated in clinical scenarios when conventional immune subsets are suppressed. Whether the subset exclusively responds during acute infection, or additionally during periods of reactivation, is also unknown. However, of relevance, a single chronically infected CMV^+^ healthy donor whose Vγ9^neg^ Vδ2^+^ subset was both clonally expanded and clearly displayed T_effector_ status was suspected to have undergone recent CMV reactivation, based on raised CMV-specific IgG levels, consistent with this latter possibility (5).

In summary, several features of the Vγ9^neg^ Vδ2^+^ T cell subset are highly suggestive of an adaptive immunobiology, which following a response can culminate in the generation of a wave of T_effector_ cells that are relatively long lived, and appear likely to provide an ongoing memory/effector contribution to immunosurveillance, most likely to chronic/recurrent infections. Ultimately this raises the intriguing possibility of whether the subset could be harnessed immunotherapeutically to enhance such protection, alongside other adaptive γδ T cell subsets such as Vδ1^+^ T cells. However, like conventional αβ adaptive immunity, the prospect that alongside protective immunity the Vγ9^neg^ Vδ2^+^ T cell subset could in some instances contribute to autoimmune responses has already been highlighted in the literature (8, 9). The recent study by Davey et al. (5) outlined here provides an intellectual and methodological basis from which to investigate the role of this intriguing new subset more fully, in both pathogen-specific immunity and immunopathological responses in different compartments of the human immune system.

## Author contributions

The ideas in this review were jointly conceived by MD, CW, SH, YO, and BW. BW wrote the first draft and all authors contributed to the final manuscript.

### Conflict of interest statement

The authors declare that the research was conducted in the absence of any commercial or financial relationships that could be construed as a potential conflict of interest.
